# MiR-152 influences osteoporosis through regulation of osteoblast differentiation by targeting RICTOR

**DOI:** 10.1080/13880209.2019.1657153

**Published:** 2019-09-06

**Authors:** Li Feng, Bo Xia, Bao-Fang Tian, Gong-Biao Lu

**Affiliations:** a Department of Traumatic Orthopedics, Jining No. 1 People’s Hospital, Jining, China;; b Department of Spine Surgery, Jining No, 1 People’s Hospital, Jining, China

**Keywords:** MicroRNA, ovariectomized rat, primary osteoblasts, MC3T3-E1 cells

## Abstract

**Context:** Evidence suggests that microRNA (miRNA) regulates gene expression and bone tissue homoeostasis of osteoporosis. MiR-152 has found to be abnormally expressed in osteoporosis, but its role in osteoblast differentiation has not been elucidated.

**Objective:** To understand the potential mechanism of miR-152 in osteoblast differentiation via regulation of RICTOR.

**Materials and methods:** The expression of miR-152 and RICTOR were tested in ovariectomized rat models of osteoporosis. Primary osteoblasts and MC3T -E1 cells were assigned into four groups, namely Control, miR-152 inhibitor, miR-control and miR-152 inhibitor + siRICTOR groups. qRT PCR and Western blots were performed to detect the expression of miR-152 and RICTOR, respectively. MTT assay was used to evaluate cell viability, and ALP activity determination and mineralization analyses were also conducted.

**Results:** In ovariectomy-induced osteoporotic rats, miR-152 (3.06 ± 0.35) in femoral tissues increased significantly, while RICTOR (0.31 ± 0.04) decreased. Compared with the Control group, the miR-152 inhibitor group presented appreciable reduction of miR-152 in primary osteoblasts and MC3T3-E1 cells, as well as remarkable increases in RICTOR, p-Akt(s473)/Akt ratio, and osteogenesis-related genes, with enhanced cell viability, ALP activity and mineralization. In comparison with cells in the miR-152 inhibitor group, those in the miR-152 inhibitor + siRICTOR group had no observable difference in miR-152, but were dramatically up-regulated in RICTOR, as well as the corresponding opposite tendencies of other factors.

**Conclusion:** Inhibiting miR-152 promoted osteoblasts differentiation and alleviated osteoporosis by up-regulating RICTOR. Therefore, miR-152 may be an essential mediator of osteoblast differentiation and a new therapeutic strategy for osteoporosis.

## Introduction

Osteoporosis is a relatively prevalent degenerative disease that mainly leads to loss of bone mass and microstructural deterioration of bone tissues and eventually increases the susceptibility of patients to bone fractures (Rachner et al. 2011). As estimated, it is more prevalent in middle-aged and elderly people, and especially, postmenopausal women had approximately over 60% incidence, posing a large threat to the health of the elderly population (Li et al. 2013; Spilmont et al. 2014). Osteoporosis has been generally believed as the consequence of osteoclast-regulated bone resorption exceeding osteoblast-regulated bone formation in the process of bone remodelling (Harvey et al. 2014). In a factual manner, the restoration and maintenance of the balance between bone formation and bone resorption constitutes an effective perspective in the clinical treatment of osteoporosis (Marie and Kassem 2011). Meanwhile, osteoblast is the major cell type of bone formation, playing important roles in metabolic balance, growth and damage repair of bone tissues (Li et al. 2015). Thus, exploring new targets for controlling osteogenesis of osteoblasts would provide new strategies and methods for the treatment of osteoporosis.

As small non-coding RNAs consisting of 18 to 25 nucleotides, microRNAs exert their regulatory function by modulating their target genes at post-transcriptional levels, and thereby taking part in various biological processes, for example, cell proliferation, differentiation, apoptosis and development (Jin et al. 2015). In many recent studies, a good number of miRNAs, such as miR-210, miR-29b, miR-125b, and miR-378, have been revealed engaged in the regulation of osteoblast differentiation by affecting the balance between bone formation and bone resorption through acting on their corresponding target genes (Mizuno et al. 2008, 2009; Kahai et al. 2009; Li et al. 2009). miR-152 is a member of the miR-148/miR-152 family and its abnormal expression has been observed in many kinds of diseases, including cancers (Liu et al. 2016). miR-148a-3p was demonstrated to inhibit osteoblast differentiation by targeting *Kdm6b* in the study of Tian et al. (2017). Kocijan et al. (2016) found the significant increase of miR-152-3p in postmenopausal patients with osteoporosis and fragile fracture, indicating a significant correlation between miR-152 and osteoporosis, which may influence the differentiation of osteoblasts, but relevant studies are rarely found. At the same time, we proved that RICTOR was a target gene of miR-152 through the target gene prediction website, and it is a key protein of the mTORC2 complex, playing an essential part in its biological function (Glidden et al. 2012). As we know, mTORC2 has great effects on cell survival, metabolism, proliferation and cytoskeleton synthesis (Kim et al. 2011). More importantly, Liu et al. (2016) found that the bone mineral density, osteoblast activity and bone resorption decreased in mice with RICTOR knockout, and the differentiation ability of osteoblasts decreased after RICTOR inhibition, suggesting an important role of RICTOR in osteoblasts and osteoporosis. But limited data were reported on whether miR-152 can target RICTOR to regulate osteoblast differentiation. Therefore, this study was conducted for the purpose of providing alternative perspectives and thoughts for the clinical prevention and treatment of osteoporosis.

## Materials and methods

### Ethics statement

The study was approved by the Ethics Committee for Laboratory Animals in our hospital and all animals in the study received standard care in compliance with the Guide for the Care and Use of Laboratory Animals published by the US National Institutes of Health (NIH Publication No. 85-23, revised 1996) (Bayne 1996).

### Establishment of ovariectomized rat model of osteoporosis

A total of 20 3-month-old healthy female Sprague-Dawley (SD) rats (weighing 280–300 g) were purchased from Shanghai SLAC Laboratory Animal Co., Ltd., and fed freely in a quiet and well-ventilated clean animal room. The rats were classified into two groups of 10 rats each in a random manner: OVX group and sham operated group (Sham). Ovariectomized (OVX) rat model of osteoporosis was constructed, and rats in OVX group were anaesthetized with 10% ketamine (100 mg/kg, Alfasan, the Netherlands) and 2% cilazin (10 mg/kg, Alfasan, the Netherlands) and fixed in the prone position. The ovaries were located near the inferior pole of the kidney through a dorsal median incision under sterile condition. The bilateral ovaries were ligated and removed with line 4, and the incisions were sutured layer by layer. In Sham group, only the same mass of adipose tissues around ovary was removed. After 12 weeks, rats were anaesthetized intraperitoneally by the combined use of ketamine and xylazine before being sacrificed. The right femora of rats were soaked in physiological saline and preserved at −20 °C for microstructure scanning with a micro-computerized tomography (micro-CT) and the detection of bone mineral density (BMD). Meanwhile, the right femora of rats were cryopreserved in liquid nitrogen at −80 °C for subsequent protein and RNA analysis.

### BMD measurement and bone histomorphometric analysis

BMD of the femur of rats was assessed with Lunar Prodigy (General Electric system, USA) after all necessary experiments that should be conducted in advance were performed. Results of BMD were given in g/cm^2^. The femur was scanned using a high-resolution Micro-Computed Tomography Scanner (SkyScan, Bruker MicroCT, Kontich, Belgium), the specimens were analysed by the Skyscan software (Dataviewer, CTVOX 2.1), and the pictures of the femur with a voxel size of 18 μm were taken at energy of 40 kV and intensity of 250 μA.

### Dual-luciferase reporter gene assay

Based on the prediction about the binding loci between human miR-152 and RICTOR, wild-type luciferase reporter gene plasmids RICTOR-wt and mutant-type RICTOR-mt plasmids which contain the RICTOR 3′UTR region were successfully constructed. Next, 293 T cells (ATCC) were cultured in 96-well plates for 23 h, with 4 × 10^4^ cells in each hole and four replicates. After substituting the culture medium with basic medium for 1 h of incubation, conventional DMEM medium was added to each hole. In accordance with the operation manual of the Lipofectamine 2000 kit (Invitrogen), the afore-mentioned luciferase reporter gene plasmids (wild-type and mutant plasmids) were unanimously transfected with miR-152 mimic/miR-control (Shanghai GenePharma Co., Ltd), and incubated for 24 h in an incubator at 37 °C. Subsequently, cells were taken out for the detection of transcriptional activity by a dual-luciferase reporter gene assay kit (Promega).

### Culture, differentiation and transfection of osteoblasts

Primary mouse calvarial osteoblastic cells were isolated by following the description in a previous study (Jeong et al. 2010). The primary osteoblasts/pre-osteoblast MC3T3-E1 cells (provided by ATCC) were firstly cultured in α-MEM medium, which contained 10% foetal bovine serum (FBS), 100 U/mL penicillin and 100 µg/mL streptomycin, in an incubator (setting: 37 °C, 5% CO_2_, and saturated humidity). In order to induce the differentiation of cells, the transfected cells were cultured with osteogenic medium (OM) supplemented by the addition of 10% FBS, 50 μg/mL ascorbic acid, and 5 mM β-glycerophosphate for 7 d. In the following, the culture medium was replaced regularly, with the frequency of once every two days. After osteogenesis induction, cells were divided into four groups, Control group, miR-152 inhibitor group, miR-control group and miR-152 inhibitor + siRICTOR group. Finally, the primary osteoblasts/MC3T3-E1 cells were inoculated into 6-well plates, following by the transfection with miR-152 inhibitors, miR-control (GenePharma) or RICTOR siRNA by using Lipofectamine 2000 kit (Invitrogen).

### qRT-PCR

Total RNA of tissues was extracted from rat osteoblasts with the application of Trizol kit (Invitrogen), which was determined for RNA purity and concentration with NanoDrop2000 (Thermo). Next, a Reverse Transcription Kit (TOYOBO, Tokyo, Japan) was used to synthesize cDNA in strict obedience to the instructions in the manufacturer’s manual. PCR primers were synthesized by Shanghai Sangon Biotech Co. Ltd (Table 1). Quantitative real-time PCR (qRT-PCR) was carried out using ABI 7500. U6 was used as the internal reference gene of miR-152. GAPDH was used as the internal reference gene for other genes and the relative expression of target genes was calculated with the application of the formula 2^-△△Ct^ method.

**Table 1.** Primers for qRT-PCR.

Gene	Sequence
miR-152	
Forward primer	5′- CGCGCTAGCAGCACGTAAAT-3′
Reverse primer	5′- GTGCAGGGTCCGAGGT-3′
U6	
Forward primer	5′-TCGCTTCGGCAGCACATATAC-3′
Reverse primer	5′-TATGGAACGCTTCACGAATTTG-3′
RICTOR	
Forward primer	5′- TGAGTACCGTGGTTCTTCTCAC-3′
Reverse primer	5′-TGCAATGGAGGGCGCTTTA-3′
ALP	
Forward primer	5′-TCAGGGCAATGAGGTCACAT-3′
Reverse primer	5′-CCTCTGGTGGCATCTCGTTA-3′
RUNX2	
Forward primer	5′-GACTGTGGTTACCGTCATGGC-3′
Reverse primer	5′-ACTTGGTTTTTCATAACAGCGGA-3′
OCN	
Forward primer	5′-CCCTGAGTCTGACAAAGCCT-3′
Reverse primer	5′-GCGGTCTTCAAGCCATACTG-3′
OSX	
Forward primer	5′-CCTCTGCGGGACTCAACAAC-3′
Reverse primer	5′-TGCCTGGACCTGGTGAGATG-3′
GAPDH	
Forward primer	5′-AACGGATTTGGTCGTATTGGG-3′
Reverse primer	5′-TCGCTCCTGGAAGATGGTGAT-3′

### MTT assay

Osteoblasts were digested by trypsin, collected in centrifugal tube, diluted to the density of 1 × 10^3^ cells/ml, and inoculated to 96-well plate for 24 h of incubation. Then, 20 μL newly prepared methyl thiazolyl tetrazolium (MTT) solution by 5 mg/mL in concentration (Sigma) was added to each well for 4 h of incubation at 37 °C, and the replacement of culture medium with 150 μL DMSO (dimethyl sulfoxide, Sigma) ensued. Four replicates were set in each group. The absorbance value (OD value) was detected at the wavelength of 490 nm with a micro-plate reader (Thermo) and cell viability was evaluated on the basis of the formula: Cell viability=(OD value of experimental group/OD value of control group)×100%.

### ALP activity and mineralization analysis

After osteoblasts were transfected with plasmids for 7 days, the activity of alkaline phosphatase (ALP) was detected by using an ALP test kit (Nanjing Jiancheng Bioengineering Institute, Nanjing, China) in accordance with the instructions in the manufacturer’s manual. After that, cells were collected, washed for two times with phosphate buffer solution (PBS), and lysed with ice-cold lysis buffer. Then, the supernatants were collected, which was incubated in SensoLyte *p*-nitrophenylphosphate at 4 °C after 15 min of centrifugation at the rate of 2500 *g*. The absorbance (OD value) was measured at 570 nm by an ELISA microplate reader (ELx800, Bio-Tek Instruments, Winooski, VT, USA). Alizarin red staining (ARS) was used to detect the mineralization ability of osteoblasts (the reagents used for staining all purchased from Nanjing Jiancheng Bioengineering Institute). Cells were cultured in 4-well plates for 24 h, added with 500 μL of 4% paraformaldehyde, kept still for 10 min, washed with PBS buffer, and added with 200 μL alizarin red dye for 30 min. Finally, cells were observed and photographed under a microscope.

### Western blot

Total protein was extracted from tissues and then determined for protein concentration with a BCA kit (Boster Biological Technology Co.Ltd). Next, loading buffer was added into the proteins for 10 min of heating at 95 °C, followed by the sample loading of 50 μg per hole. Electrophoresis with 10% polyacrylamide gel (Boster Biological Technology co.ltd) was used to separate proteins, which were transferred to the PVDF membrane and blocked for 1 h with 5% BSA at room temperature. Next, primary antibodies (1:500 diluted) were added for overnight reaction at 4 °C, including RICTOR (ab70374, Abcam), p-Akt (s473) (ab8932, Abcam), Akt (ab8805, Abcam), and GAPDH (ab181602, Abcam). The membrane with proteins on it was washed for three times, 5 min each time, before the addition of secondary antibodies (1:1000 diluted, Abcam) for 1 h of incubation at room temperature. Subsequently, the membrane was washed again for three times/5 min before the development with ECL chemiluminescence reagent. With GAPDH as the internal reference gene, the gray value of target bands was analysed with the software Image J.

### Statistical methods

The statistical software SPSS 21.0 was used for quantitative data analysis and each experiment was separately conducted for three times to get the mean value and reduce errors. Measurement data were presented by mean ± standard deviation ($\bar{x}$ ± s). The comparison between two groups was performed by using Student’s *t*-test, while difference among multiple groups was analysed by using one-way ANOVA. *Post hoc* test was performed by Tukey’s method. *p* < 0.05 indicated the statistical significance of differences.

## Results

### Expression of miR-152 and RICTOR in ovariectomized rat model of osteoporosis

**Figure 1.**
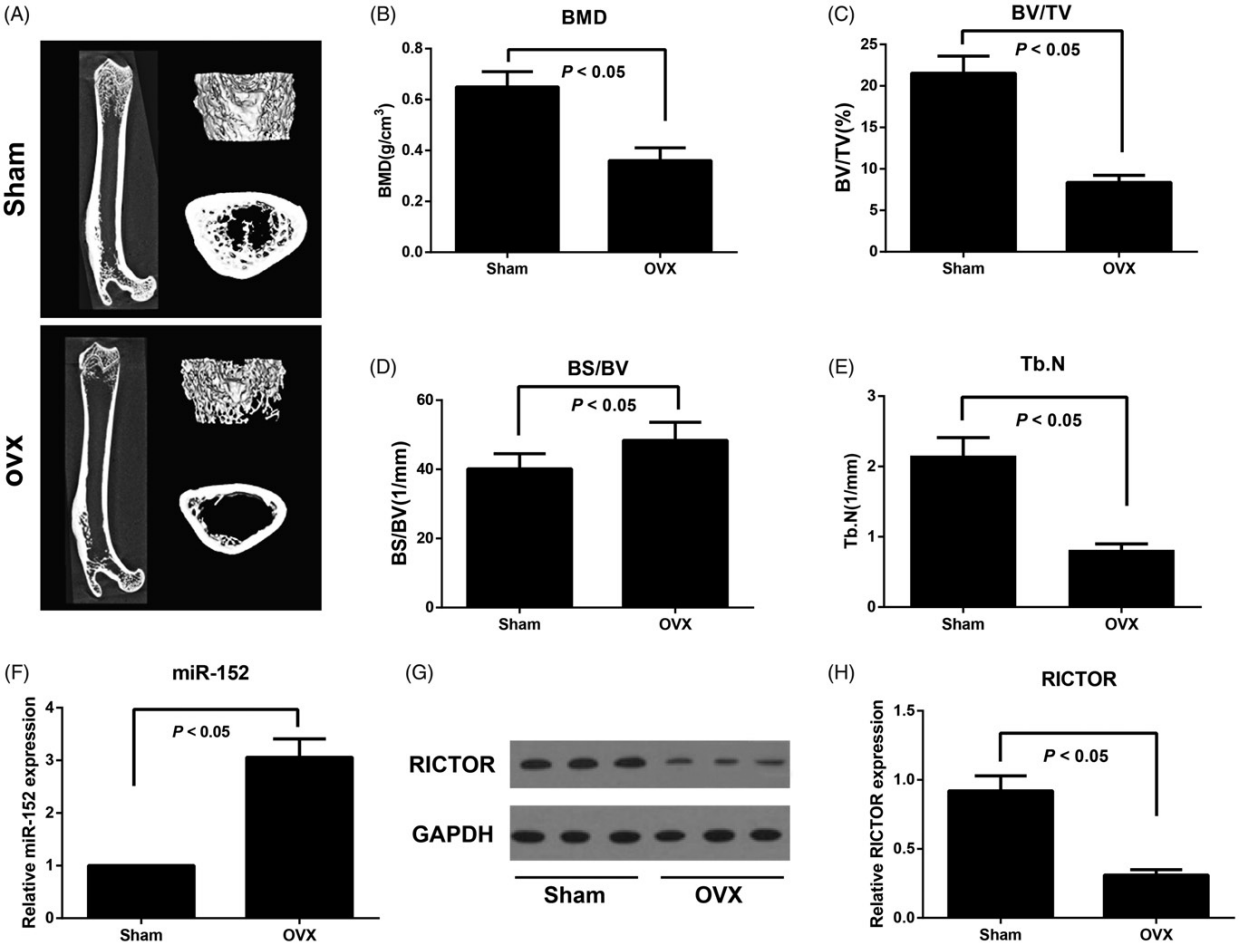
Bone microarchitectural changes in femur assessed by micro-CT and expression of miR-152 and RICTOR in ovariectomized rat model of osteoporosis. Note: A, Representative micro-CT images of the right femur; B, Bone mineral density (BMD) of femoral tissues was measured; C–E, Micro-CT analysis of BV/TV (C), BS/BV (D), and Tb.N (E) in both OVX and Sham rats; BV/TV, Bone volume/total volume; BS/BV, Bone surface/bone volume; Tb.N, trabecular number; F, The miR-152 levels in femoral tissues in both OVX and Sham rats detected by qRT-PCR; G, H, RICTOR protein expression in femoral tissues in both OVX and Sham rats detected by Western blot.

As shown in Figure 1, rats in the OVA group declined significantly in BMD, BV/TV and Tb.N levels and went up appreciably in BS/BV levels compared to those in the Sham group (all *p* < 0.05), suggesting that the ovariectomized rat model of osteoporosis had been successfully established. By detecting the expression level of miR-152 and RICTOR in femoral tissues, we found rats in OVA group had higher miR-152 and lower RICTOR expression than those in Sham group (both *p* < 0.05).

### RICTOR: the target gene of miR-152

**Figure 2.**
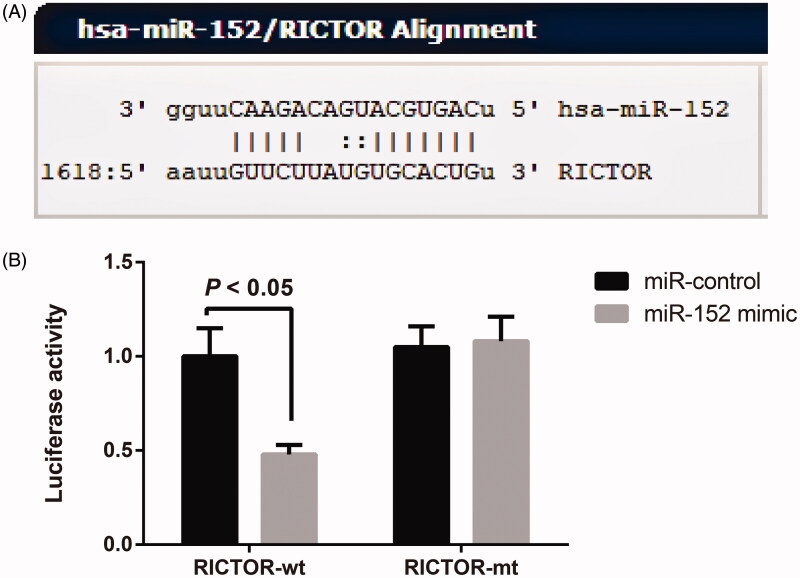
RICTOR was the target gene of miR-152. Note: A, The complementary binding sequence of RICTOR to miR-152 shown in the target gene prediction website; B, Dual-luciferase reporter gene assay verified the targeting relationship between RICTOR and miR-152.

By retrieving and analysing the data on the target gene prediction website microrna.org (http://www.microrna.org/microrna/home.do), we concluded that the 3′UTR region of RICTOR contains a region complementary with and able to bind to miR-152 (Figure 2(A)). According to the results of dual-luciferase reporter gene assay, in RICTOR-wt groups, cells transfected with miR-152 mimic were significantly lower in luciferase activity than those transfected with miR-control (all *p <* 0.05); however, in RICTOR-mt groups, transfection with either miR-152 mimic or miR-control could significantly change the luciferase activity of cells (all *p <* 0.05, Figure 2(B)). These findings supported the conclusion that RICTOR was a target gene of miR-152.

### Expression of osteogenesis-related genes in each group

**Figure 3.**
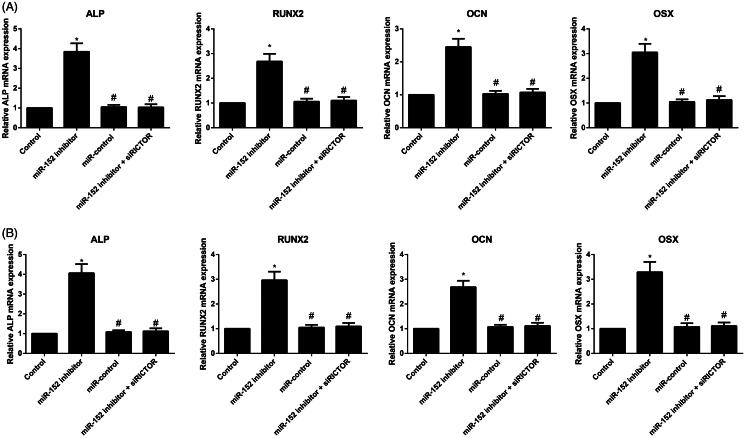
The mRNA expression of *ALP, RUNX2, OCN* and *OSX* in primary osteoblasts (A) and MC3T3-E1 cells (B) detected by qRT-PCR. Note: *p< 0.05 compared with Control group; #*p<* 0.05 compared with miR-152 inhibitor group.

Compared with the Control group, primary osteoblasts and MC3T3-E1 cells in the miR-152 inhibitor group were remarkably increased in mRNA expression of *ALP, RUNX2, OCN* and *OSX* (all *p* < 0.05), while those in the miR-control group showed no observable difference in these four indexes (all *p* > 0.05, Figure 3). Besides, cells in the miR-152 inhibitor + siRICTOR group had down-regulated *ALP, RUNX2, OCN* and *OSX* mRNAs in comparison with the miR-152 inhibitor group (all *p* < 0.05).

### Comparison of cell viability and ALP activity of osteoblasts

**Figure 4.**
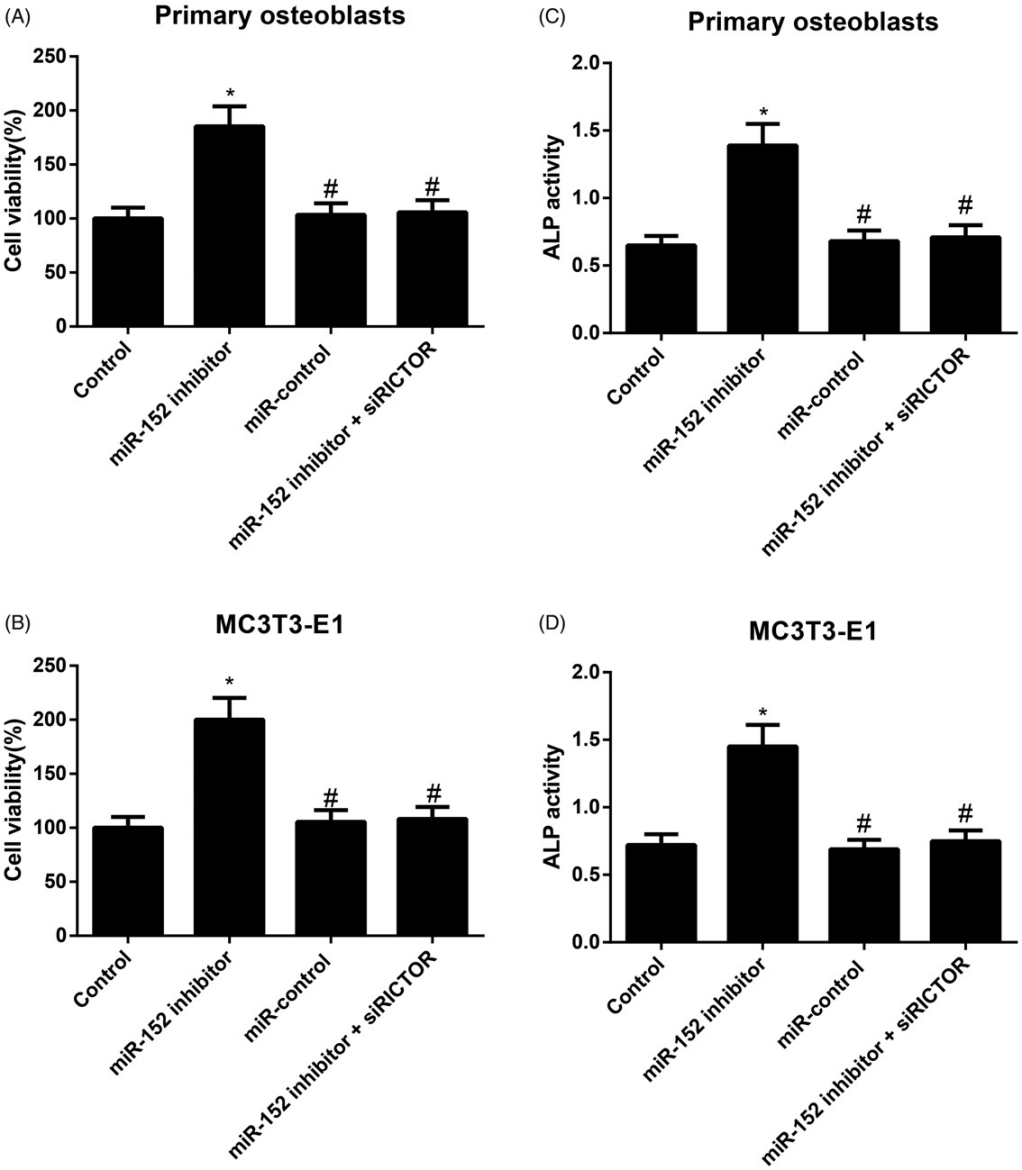
Comparison of the viability and ALP activity of osteoblasts in different groups. Note: A, B, Cell viability of primary osteoblasts (A) and MC3T3-E1 cells (B) in each group detected by MTT assay; C, D, ALP activity of primary osteoblasts (C) and MC3T3-E1 cells (D) in each group; *p< 0.05 compared with Control group; #*p<* 0.05 compared with miR-152 inhibitor group.

As shown in Figure 4, when compared to the primary osteoblasts and MC3T3-E1 cells in Control group, those in the miR-152 inhibitor group dramatically increased in the survival rate of cells and the activity of ALP, but those in the miR-control group demonstrated no statistical difference in regard to the two indexes (all *p* > 0.05). Besides, these cells had lower cell viability and ALP activity in miR-152 inhibitor + siRICTOR group than those in the miR-152 inhibitor group (both *p* < 0.05).

### Comparison of mineralized nodules of osteoblasts

**Figure 5.**
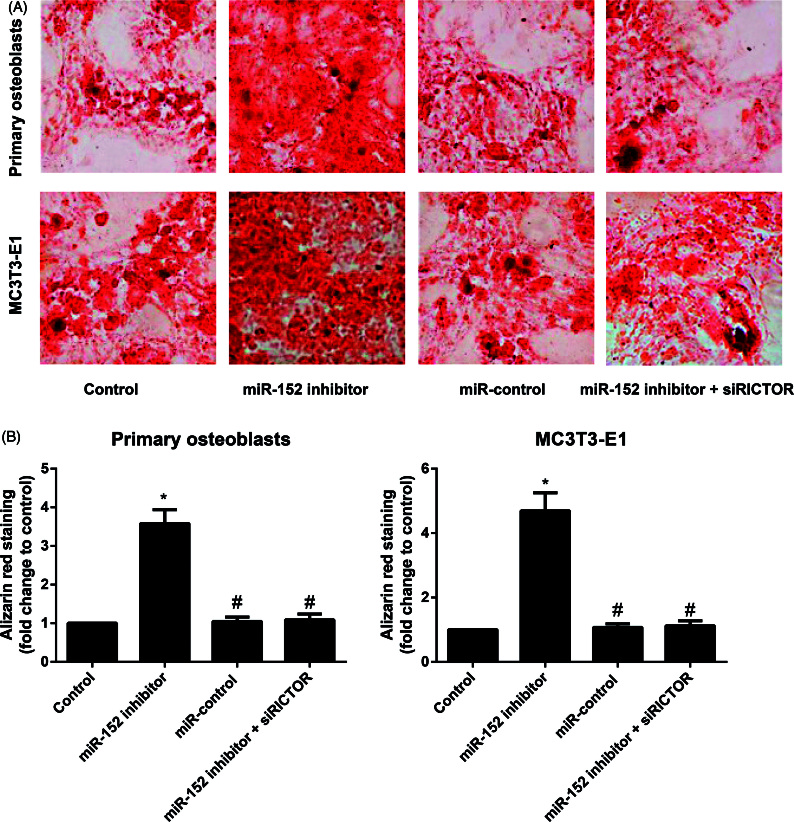
Mineralized nodules of primary osteoblasts and MC3T3-E1 cells in each group. Note: A, Mineralized nodules of primary osteoblasts and MC3T3-E1 cells in each group examined after Alizarin red staining; B, Quantitative analysis of alizarin red staining results in primary osteoblasts and MC3T3-E1 cells; *p< 0.05 compared with Control group; #*p<* 0.05 compared with miR-152 inhibitor group.

Reddish orange mineralized nodules were observed under the microscope after alizarin red staining. According to statistical results, miR-152 inhibitor group had appreciably increased mineralized nodules in primary osteoblasts and MC3T3-E1 cells by comparison with Control group (all *p* < 0.05, shown in Figure 5), while miR-control group demonstrated no observable difference in mineralized nodules (all *p* > 0.05). Meanwhile, cells in the miR-152 inhibitor + siRICTOR group had reduced mineralized nodules relevant to those in the miR-152 inhibitor group (*p* < 0.05).

### Expression of miR-152, RICTOR and downstream proteins in osteoblasts

**Figure 6.**
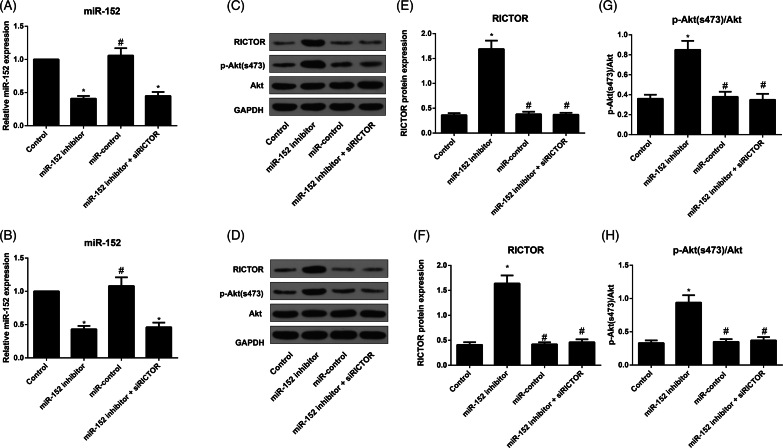
Comparison of expression of miR-152, RICTOR and downstream proteins in osteoblasts from each group. Note: A, B, The expression of miR-152 in primary osteoblasts (A) and MC3T3-E1 cells (B) detected by qRT-PCR; C, D, The expression of RICTOR and p-Akt (s473)/Akt in primary osteoblasts (C) and MC3T3-E1 cells (D) evaluated by Western blot; E, F, The expression of RICTOR in primary osteoblasts (E) and MC3T3-E1 cells (F) in each group; G, H, Comparison of p-Akt(s473)/Akt levels in primary osteoblasts (G) and MC3T3-E1 cells (H) among groups; *p< 0.05 compared with Control group; #*p<* 0.05 compared with miR-152 inhibitor group.

To detect and analyse the expression levels of miR-152, RICTOR and downstream proteins in primary osteoblasts and MC3T3-E1 cells, we used the technology of qRT-PCR and Western blot according to standard operation procedures proved effective by many previous researchers (Figure 6). As compared with the Control group, miR-152 inhibitor group showed remarkable reduction in miR-152, and significant up-regulations of RICTOR and p-Akt(s473)/Akt ratio (all *p* < 0.05). However, miR-152 inhibitor + siRICTOR group showed no significant difference in miR-152 from miR-152 inhibitor group, with significantly decreased levels of RICTOR and p-Akt (s473)/Akt (all *p* < 0.05).

## Discussion

In this study, we successfully established the ovariectomized rat model of osteoporosis and found the significant reduction of BMD, BV/TV and Tb.N levels, with apparent elevation of BS/BV in rats, which was consistent with the finding by a previous study (Hao et al. 2016). To our knowledge, BMD, BS/BV and BV/TV were generally considered as bone mass-related indicators (Wedemeyer et al. 2007), and Tb.N was used to reflect trabecular bone dispersion and spatial structure (Chiba et al. 2012). Obviously, the changes of the above indicators in ovariectomized rats suggested the decreased bone mass, thinned thickness and decreased number of trabecular, widened gap, degenerated skeletal structure, and the reduced bearing capacity, in turn indicated the successfully established models of osteoporosis (Kang et al. 2013). In the meantime, a decreased miR-152 and increased RICTOR were also discovered in ovariectomized rat model with osteoporosis in our detection. Similar to our results, Kocijan et al. (2016) also shed light upon the significantly increased miR-152-3p expression in patients suffering from postmenopausal osteoporosis and fragile fracture, and RICTOR expression was reduced gradually in aged osteoporotic mice, as analysed by Pinling Lai et al. (2016). Hence, these findings suggested the abnormal expression of miR-152 and RICTOR in osteoporosis.

In addition, we also cultured the osteoblasts *in vitro* and had them transfected with desired plasmids. Consequently, we observed the effectively increased the viability of primary osteoblasts and MC3T3-E1 cells after treated with miR-152 inhibition. A similar study analysed another member of miR-148/miR-152 family, miR-148a-3p, which could inhibit the growth and activity of osteoblasts to further inhibit osteoblast differentiation via down-regulating its target gene *Kdm6b* (Tian et al. 2017). As a matter of fact, there is also a study demonstrating that most osteoblasts are derived from bone marrow mesenchymal stem cells and their differentiation process is apparently affected by the modulation of many transcription factors and cytokines (Hartmann 2006). ALP, as one of the early indicators of osteoblast differentiation, could represent the state of bone formation, since its expression increased with the development of cell differentiation (Singhatanadgit and Olsen 2011). OCN, an indispensable substance for bone mineralization, has been believed as a medium-term indicator of osteoblast differentiation, and it binds to crystals and deposits in collagen fibers to increase the mineralization of bone matrix (Kosan et al. 2012). Runx2 was preferentially expressed in osteoblasts and it makes a key transcription factor influencing the differentiation of osteoblasts and the process of bone formation (Komori 2006; Maruyama et al. 2007). Osx is a kind of transcription factor with zinc finger structure and an indispensable osteoblast-specific transcription factor (Nakashima et al. 2002). After detecting the specific differentiation markers and transcription factors of osteoblasts in our experiments, the miR-152 inhibitor was found to promote the expressions of *ALP, RUNX2, OCN* and *OSX* genes, indicating that inhibiting miR-152 may promote early osteoblasts differentiation via enhancing the transcription of differentiation markers of osteoblasts. Additionally, alizarin red staining was performed in order to observe the formation of mineralized nodules, which can be very informative as the final expression form of osteoblastic phenotype *in vitro* (Ferre et al. 2014). So, another important finding of this study is that miR-152 inhibitor can appreciably augment the number of mineralized nodules of osteoblasts, which advances the maturation and mineralization of osteoblasts through the hindrance of miR-152 expression. It is worth mentioning that siRICTOR can counteract the effect of miR-152 inhibitor on promoting osteoblast differentiation in our models. Consistent evidence from Pinling Lai et al. (2016) added weight to the conclusion by proving that miR-218 could reduce the surface adhesion and viability of osteoblasts by directly and specifically inhibiting the expression of RICTOR, which can ultimately lead to the reduction in the number of functional osteoblasts and an acceleration of bone loss in aging mice, which suggested the regulatory role of miR-152 on RICTOR.

To further explore the action between miR-152 and RICTOR, firstly, we confirmed by dual-luciferase reporter gene assay in our work that RICTOR was a target gene of miR-152, which was in line with the finding of Tsuruta et al. (2011) in endometrial cancer. Secondly, the expression of RICTOR and p-Akt (s473)/Akt level were determined by qRT-PCR and Western blot to be significantly increased by miR-152 inhibitor. In general, mTOR signalling pathway activates protein translation and regulates protein synthesis, acting as the central regulator of cell proliferation, growth and differentiation (Sarbassov et al. 2005). As a core component, mTOR forms two complexes with other different proteins, including mTORC1 and mTORC2 (Huang and Fingar 2014). As a special member of mTORC2 molecules, RICTOR can bind to mTOR to induce the direct phosphorylation of Ser473 site of RICTOR in mTORC2-mediated signalling pathway, thus activating AKT and promoting cell survival and proliferation (Razmara et al. 2013). p-AKT (s473) is the downstream of RICTOR and a classic pathway for cell survival, whose down-regulation would effectively affect cell survival (Breuleux et al. 2009). More importantly, several previous studies have proved that AKT-mTOR signaling pathway can effectively regulate the transcription process of osteogenesis-related genes, including Runx2 (Shoba and Lee 2003; Pantovic et al. 2013). In the meantime, the cytoskeleton changes and osteogenic differentiation of cells could be inhibited after RICTOR interference (Martin et al. 2015). For example, the activity and differentiation of osteoblasts decreased significantly after RICTOR deletion, thus promoting the occurrence of osteoporosis (Liu et al. 2016). Besides, the specific knockout of RICTOR in osteoblasts can inhibit the expression of p-Akt (s473) and RUNX2 and promote the occurrence of osteoporosis in the elderly mice (Lai et al. 2016). Taken together, miR-152 inhibitor may activate p-AKT (s473) by up-regulating its target gene RICTOR, enhance the activity of osteoblasts and promote the expression of downstream osteoblast marker genes, thereby promoting osteogenic differentiation and maturation.

## Conclusions

An increased miR-152 and decreased RICTOR were found in osteoporosis. Importantly, inhibiting miR-152 could up-regulate the expression of its target gene RICTOR to improve the viability of osteoblasts and promote osteoblast differentiation, which is expected to provide alternative strategies and research perspective for the prevention and treatment of osteoporosis.
